# Otolith chemical fingerprints of skipjack tuna (*Katsuwonus pelamis*) in the Indian Ocean: First insights into stock structure delineation

**DOI:** 10.1371/journal.pone.0249327

**Published:** 2021-03-29

**Authors:** Iraide Artetxe-Arrate, Igaratza Fraile, Jessica Farley, Audrey M. Darnaude, Naomi Clear, Naiara Rodríguez-Ezpeleta, David L. Dettman, Christophe Pécheyran, Iñigo Krug, Anaïs Médieu, Mohamed Ahusan, Craig Proctor, Asep Priatna, Pratiwi Lestari, Campbell Davies, Francis Marsac, Hilario Murua

**Affiliations:** 1 AZTI, Marine Research, Basque Research and Technology Alliance (BRTA), Pasaia, Gipuzkoa, Spain; 2 CSIRO Oceans and Atmosphere, Hobart, Tasmania, Australia; 3 Marbec, Univ Montpellier, CNRS, Ifremer, IRD, Montpellier, France; 4 Environmental Isotope Laboratory, Department of Geosciences, University of Arizona, Tucson, Arizona, United States of America; 5 Université de Pau et des Pays de l’Adour, E2S UPPA, CNRS, IPREM, Pau, France; 6 Marbec, Univ Montpellier, CNRS, Ifremer, IRD, Victoria, Seychelles; 7 Maldives Marine Research Institute, Ministry of Fisheries, Marine Resources and Agriculture, Male, Maldives; 8 Research Institute for Marine Fisheries, Jakarta, Indonesia; 9 Marbec, Univ Montpellierm CNRS, Ifremer, IRD, Sète, France; 10 International Seafood Sustainability Foundation, Washington, DC, United States of America; Universidad de Cádiz, Facultad de Ciencias del Mar y Ambientales, SPAIN

## Abstract

The chemical composition of otoliths (earbones) can provide valuable information about stock structure and connectivity patterns among marine fish. For that, chemical signatures must be sufficiently distinct to allow accurate classification of an unknown fish to their area of origin. Here we have examined the suitability of otolith microchemistry as a tool to better understand the spatial dynamics of skipjack tuna (*Katsuwonus pelamis*), a highly valuable commercial species for which uncertainties remain regarding its stock structure in the Indian Ocean. For this aim, we have compared the early life otolith chemical composition of young-of-the-year (<6 months) skipjack tuna captured from the three main nursery areas of the equatorial Indian Ocean (West, Central and East). Elemental (Li:Ca, Sr:Ca, Ba:Ca, Mg:Ca and Mn:Ca) and stable isotopic (δ^13^C, δ^18^O) signatures were used, from individuals captured in 2018 and 2019. Otolith Sr:Ca, Ba:Ca, Mg:Ca and δ^18^O significantly differed among fish from different nurseries, but, in general, the chemical signatures of the three nursery areas largely overlapped. Multivariate analyses of otolith chemical signatures revealed low geographic separation among Central and Eastern nurseries, achieving a maximum overall random forest cross validated classification success of 51%. Cohort effect on otolith trace element signatures was also detected, indicating that variations in chemical signatures associated with seasonal changes in oceanographic conditions must be well understood, particularly for species with several reproductive peaks throughout the year. Otolith microchemistry in conjunction with other techniques (e.g., genetics, particle tracking) should be further investigated to resolve skipjack stock structure, which will ultimately contribute to the sustainable management of this stock in the Indian Ocean.

## Introduction

Skipjack tuna (*Katsuwonus pelamis*) is a cosmopolitan species inhabiting tropical and subtropical waters of the Indian, Pacific and Atlantic Oceans [1]. This species is, by far, the most commonly caught tuna worldwide [2, 3]. Currently, skipjack tuna stocks are considered to be in a healthy status in all oceans, both in terms of stock abundance and fishing mortality [3]. Five stocks of skipjack tuna are considered globally for management, two in the Atlantic Ocean, two in the Pacific Ocean and one in the Indian Ocean. Regional studies indicate a more complex stock structure than currently assumed for assessment and management purposes of skipjack tuna in the Indian Ocean [4, 5]; however, lack of understanding of population dynamics and connectivity at oceanic scale do not allow separation of stocks [6].

Stock structure understanding is essential to determine a suitable spatial scale for management, as the way a stock will respond to management decisions cannot be accurately predicted if the boundaries that characterize a stock are not correctly defined [7]. Skipjack tuna spawns throughout the year in large warm water tropical areas, but as those areas are often poor feeding grounds, they move towards adjacent colder and more productive subtropical areas to feed [8–10]. However, not all skipjack tuna seem to perform these long-distance movements, but rather, some remain around the tropical spawning area [10, 11]. As a result, the spatial dynamics of skipjack tuna and, hence, its implications on the stock structure of the species needs to be evaluated. Besides, skipjack tuna are fast growing, early maturing species, and have high reproductive potential, which makes this species more resilient to fishing pressure than many other tuna species [12–14]. However, fishing pressure may vary spatially due to factors such as fleet operational constrains, variations in population densities, and differences in the value of fish caught, among others [15]. Thus, excessive local fishing effort and catches, may lead to situations of local overfishing and depletion when skipjack movements between areas are limited [16]. The understanding of exchange rates and connectivity among recruits from different regions is therefore, essential to achieve a sustainable and effective management of this species [17].

There are several methods that can be used to study fish stock structure, which can provide information at different spatial and/or temporal scales [18]. Among them, analyses of otolith (i.e., calcified structures found in the inner ear of the fish) chemical composition is widely used to explore movements and habitat use of fish [19]. This method relies on the premise that ambient water chemistry and environmental conditions (but also other intrinsic factors such as physiology, diet or genetics) affect the elemental incorporation (at minor and trace quantities) into the concentric growth of the otolith during daily increment formation [20, 21]. Since otoliths are both acellular and metabolically inert, material accreted during otolith formation is preserved as fish grows [22]. As such, the chemical composition of the material accreted during early life stages, serves as a natural marker to identify fish that have inhabited environments with distinct physicochemical characteristics [23]. Otolith microchemistry proved to be a powerful tool to discriminate among nursery fish groups for other tropical tuna species [24–26], and also to depict different movements and life history patters of skipjack tuna in the Pacific Ocean [27].

Here, we examined otoliths of young-of-the-year (YOY) skipjack tuna collected across the equatorial strip of the Indian Ocean, where high larvae concentrations and spawning activity of this species have been observed; off Seychelles, Somalia and Mozambique Channel in the western Indian Ocean, off Maldives in the central Indian Ocean, and off Sumatra in the eastern Indian Ocean [9, 28, 29]. Besides, those areas are also important fishery grounds of skipjack tuna in the Indian Ocean [6]. Our aim was to determine whether YOY skipjack tuna captured within these three regions could be spatially discriminated based on their early life otolith microchemistry composition. If so, the characterized nursery origin signature can then be used as a baseline sample to predict older skipjack tuna origin, by analyzing the same early life portion of the otolith [30, 31]. This in turn, will contribute to improve our understanding of the connectivity and mixing rates and, ultimately, the stock structure of this species in the Indian Ocean.

## Materials and methods

### Fish sampling

**Fig 1:**
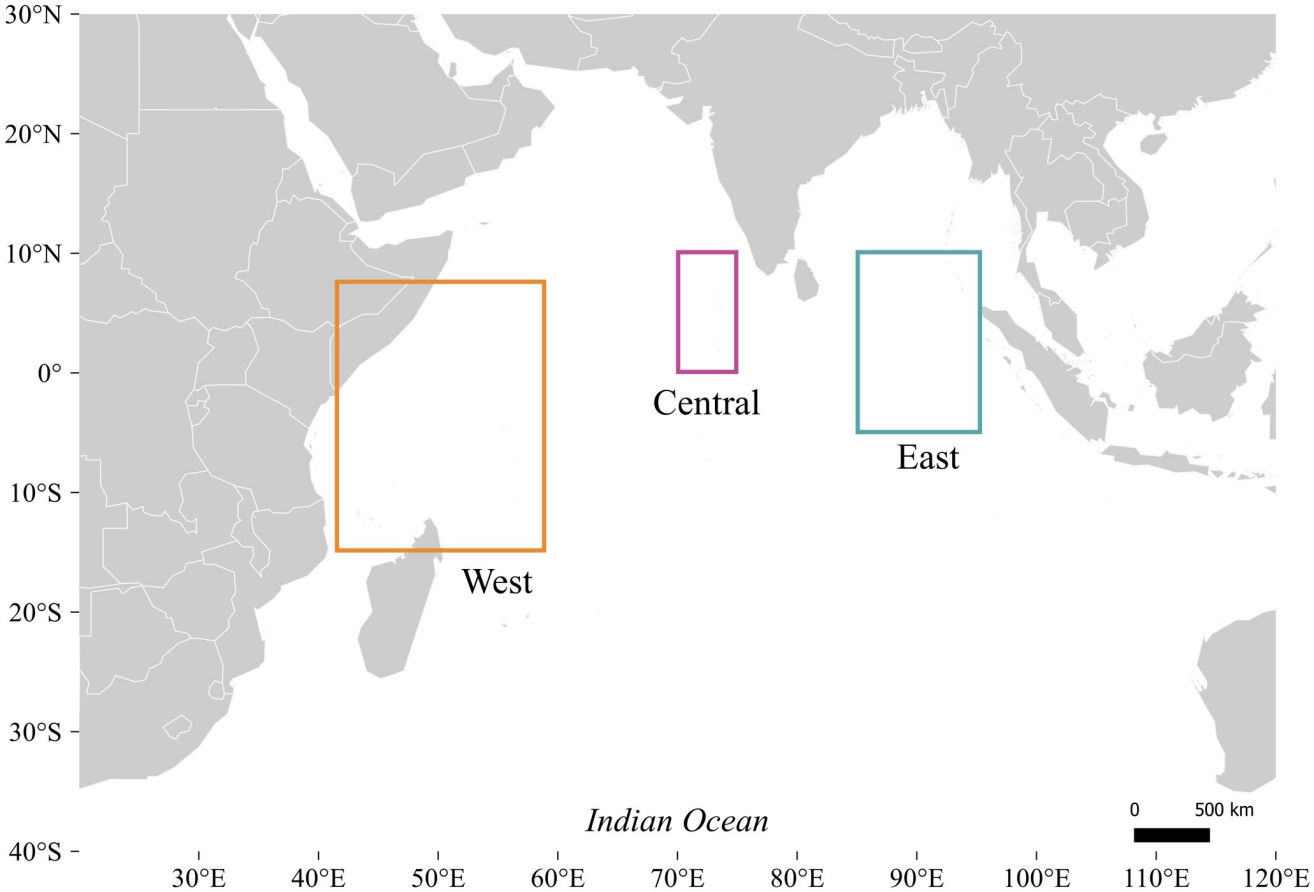
Skipjack tuna (*Katsuwonus pelamis*) sampling locations across the equatorial Indian Ocean. Squares represent nursery areas where YOY skipjack tuna were captured from, referred to as West (orange), Central (purple) and East (green).

Skipjack tuna were collected from three distinct nursery areas in the Indian Ocean: West (10°S-10°N, 40°E-60°E), Central (0°-10°N, 65°E-75°E), and East (5°S-10°N, 85°E-95°E) (Fig 1).

Samples were obtained directly by scientist or scientific observers on-board purse seine vessels or at port during two consecutive years (2018 and 2019), as part of a collaborative research project on Population Structure of Tuna, Billfish and Sharks of the Indian Ocean [32]. Fish size ranged between 24.5 and 35.0 cm fork length (FL) (Tables 1 and 2), and were estimated to be about 4–6 months old following the age-length relationship described by Eveson et al. [33]. Thus, we assumed that capture locations represent nursery areas where fish reside during the early juvenile stage. Note that fish were sampled at different years/time periods, which implies that the otolith collection available for this study comprised fish from different cohorts (S1 Fig). Those spawned from mid (August) 2017 to early (April) 2018 were assigned to the 2017 cohort, hereafter “2017”. Those spawned from mid (May) 2018 to early (January) 2019 were assigned to the 2018 cohort, hereafter “2018”.

**Table 1:** Number, size, sampling period and mean values of element: Ca ratios measured in otoliths of skipjack tuna (*Katsuwonus pelamis*) at each of the three nursery areas sampled.

Nursery	Cohort	N	FL range	Sampling period	Li:Ca (x10^-6^)	Sr:Ca (x10^-3^)	Ba:Ca (x10^-6^)	Mg:Ca (x10-4)	Mn:Ca (x10-6)
West	2017	30	29.0–35.0	Mar 2018	1.18±0.03	3.92±0.06	3.11±0.21	1.16±0.06	6.03±0.42
West	2018	21	29.0–33.0	Apr 2019	1.16±0.05	4.23±0.10	4.19±0.51	1.41±0.06	5.70±0.44
Central	2017	18	28.0–34.0	Aug 2018	1.15±0.05	4.24±0.10	4.03±0.56	1.32±0.06	6.59±0.48
Central	2018	18	28.5–33.0	Feb 2019	1.18±0.05	4.55±0.14	7.00±1.44	1.21±0.06	7.58±0.92
East	2017	20	25.0–33.5	Apr 2018	1.17±0.04	4.28±0.09	6.53±1.01	1.45±0.09	5.17±0.48
East	2018	21	24.5–35.0	Nov 2018	1.13±0.05	4.26±0.16	4.69±0.56	1.34±0.09	8.12±0.87

Size is fork length (FL) in cm. N, number of fish analyzed.

**Table 2:** Number, size, sampling period and of carbon and oxygen stable isotope ratios mean values measured in otoliths of skipjack tuna (*Katsuwonus pelamis*) at each of the three nursery areas sampled.

Nursery	Cohort	N	FL range	Sampling period	δ^13^C	δ^18^O
West	2017 and 2018	20	29.0–35.0	Mar 2018 and Apr 2019	-8.74±0.09	-1.35±0.06
Central	2017 and 2018	18	29.0–33.0	Aug 2018 and Feb 2019	-8.72±0.13	-1.61±0.06
East	2017 and 2018	18	24.5–35.0	Apr and Nov 2018	-8.80±0.10	-1.78±0.05

Size is fork length (FL) in cm. N, number of fish analyzed.

### Otolith preparation and analyses

Sagittal otoliths were extracted, cleaned of adhering organic tissue, rinsed with ultrapure water (Milli-Q), and stored dry in plastic vials. In the laboratory, one otolith of each specimen was embedded in two-part epoxy resin (Araldite 2020, Huntsman Advanced Materials, Switzerland). Blocks were polished using 3M^®^ silicon carbide sandpaper (particle size = 220 μm) and a lapping wheel with a series of decreasing grain diameter (30, 15, 9, 3 and 1 μm) 3M^®^ silicon carbide lapping discs, moistened with ultrapure water, to obtain a transverse section where the core was exposed. Sections were ultrasonically cleaned using ultrapure (Milli-Q) water for 10 minutes. Following sonication, otolith sections were left to air dry in loosely capped vials for 24 h before being glued in a sample plate using Crystalbond thermoplastic glue (Crystalbond 509; Buehler). When a single otolith was available only trace element analyses were conducted. When both pairs of otoliths were available, the second one was used for carbon and oxygen stable isotope analyses.

#### Trace element analyses

Otoliths (n = 128) were analysed for trace element chemistry using a high resolution inductively coupled plasma mass spectrometer (HR-ICP-MS, Element XR, Thermo Scientific, Bremen, Germany), coupled to a high repetition rate 1030 nm femtosecond laser (fs-LA) system (Alfamet, Neseya, Canejan, France) available at the Institut des Sciences Analytiques et de Physico-Chimie pour l’Environnement et les Matériaux, Université de Pau et des Pays de l’Adour/CNRS (Pau, France). Each otolith was ablated 200 μm apart from the primordium along the ventral arm (S2 Fig). This spot was considered to be representative of the chemical signature corresponding to the first 13–15 days of life of the fish according to direct daily microincrement counts, and thus to exclude the potential maternal effects the primordium (i.e., the initial structure of an otolith) may incorporate [34]. Laser ablation conditions were 200 Hz, a laser beam size of 15 μm and 30 μJ per pulse energy corresponding to a fluence of 14 J/cm2, until the depth limit of ablation (< 30 μm). During ablation the small laser beam was continuously and rapidly moved (500 μm s-1) at the surface of the sample due to a 2D galvanometric scanner in order to create a trajectory made of 6 concentric circles [35]. This resulted in the ablation of a crater 30 μm in diameter and 30 μm deep. The ablation cell was flushed with argon to transport laser-induced particles to the HR-ICP-MS. The fsLA-HR-ICP-MS was tuned daily to reach optimal particle atomization conditions and minimal elemental fractionation. This was obtained for a U/Th signal ratio of 1 ± 0,05 using NIST 612. The mass spectrometer was used in the medium-resolution mode (R = 4000) to ensure a complete polyatomic interference removal for the isotopes of interest. Relative abundances of five isotopes (^7^Li, ^88^Sr, ^138^Ba, ^24^Mg and ^55^Mn) were estimated, as well as ^43^Ca, which was used as the internal standard. The concentration of ^43^Ca in the otolith was assumed to be constant at 38.3% [36]. Data reduction including background subtraction, conversion to ppm and standardization to calcium (element:Ca μmol mol-1) was done using an in-lab developed software FOCAL 2.27. National Institute Standards and Technology (NIST) 610 and 612 glass standards with known chemical composition were used for calibration. Measurement accuracy was determined based on an otolith certified reference material for trace elements (FEBS-1, NRC-CNRC, Canada). To correct for short-term instrumental drift, standards and reference material were measured threefold two times a day; at the beginning and the end of each session. Trace element measurements of the blank sample gases were recorded for 20-30s before each sample ablation of ~40s. Mean relative standard deviation (RSD) for NIST 612 and 610 were (n = 9): 2.9% and 8.5% (Li), 2.1% and 8.9% (Sr), 1.8% and 4.8% (Ba), 2.5% and 9.4% (Mg), 2.3% and 3.7% (Mn), respectively. The elemental ratios of Li:Ca, Sr:Ca, Ba:Ca, Mg:Ca and Mn:Ca exceeded the detection limits of the fs-LA-ICP-MS for all samples.

#### Stable isotopes analyses

For stable isotope analyses (n = 56), microsampling of otolith powder for carbon (δ^13^C) and oxygen (δ^18^O) stable isotope analysis was performed using a high-resolution computerised micromill (New Wave MicroMill System, NewWave Research G. C. Co., Ltd, Cambs, UK). The area of analysis on the smallest skipjack tuna otolith (24.5 cm FL) was used to create a standard template that was then applied to the remaining otoliths, to ensure that the same portion of the otolith was analyzed in every fish (S2 Fig). Therefore, the drill path covered the area of the otolith corresponding to the first ~4 months of life (according to Eveson et al. [33] age-length relationship), with a larger time period of the otolith sampled for stable isotopes than trace elements due to differences in sample material requirements. Ten drill passes were run at a depth of 50 μm per pass over a preprogrammed drill path using a 300-μm diameter carbide bit (Komet dental; Gebr. Basseler, Lemgo, Germany). Powdered material was then analysed for δ^13^C and δ^18^O on an automated carbonate preparation device (KIEL-III, Thermo- Fisher Scientific, Waltham, MA, USA) coupled to a gas-ratio mass spectrometer (Finnigan MAT 252, ThermoFisher Scientific) at the Environmental Isotope Laboratory of the University of Arizona. All isotope values were reported according to standards of the International Atomic Energy Agency in Vienna. The isotope ratio measurement was calibrated based on repeated measurements of NBS-19 and NBS-18 (International Atomic Energy Agency standards). Measurement precision was ± 0.10 ‰ for δ^18^O and ±0.08‰ for δ^13^C (1 sigma).

### Statistical analyses

All statistical analyses were performed using open access R software [37]. Prior to all multivariate analyses, otolith chemistry data was standardized (i.e., for each element, the data was centered by subtracting the mean and scaled by dividing by the standard deviation) to give the same weight to all elements and stable isotopes.

Elements were first analyzed individually. Parametric assumptions were violated by Ba:Ca, Mg:Ca and Mn:Ca. Therefore, non-parametric tests were used for trace element analyses, to apply a consistent approach for all elements. To determine whether the otolith chemistry of YOY skipjack tuna varied spatially and/or temporally, a permutational multivariate analysis of variance (PERMANOVA) was used to test for differences in element:Ca ratios between nurseries and cohorts [38, 39]. Nursery and cohort were fixed factors in the full factorial model. The resemblance matrix was based on euclidean distance dissimilarities and the number of unrestricted permutations was set to 999 random repeats using *adonis* {vegan}. Statistical significance was determined based on adjusted p-values after the Benjamini-Hochberg correction [40], and post hoc pairwise comparisons were applied to identify the source of differentiation between group means using *lincon* {WRS2}. Parametric assumptions of normality and homoscedasticity were met for δ^13^C and δ^18^O data, and therefore one-way ANOVA test was used for comparisons among nurseries, *aov* {stats}. When significant differences were detected, post hoc comparison were performed to the source of differences between means, using *TukeyHSD* {stats}.

Both trace elements and stable isotopes were them combined for multivariate analyses. The resultant dataset was limited by the number of individuals for which both types of data were available (n = 56). A multilevel pairwise comparison was performed using *pairwise*.*adonis* {pairwiseAdonis} to test for differences in the multielemental signature between nursery areas. The resemblance matrix was based on euclidean distance dissimilarities and the number of unrestricted permutations was set to 999 random repeats. Statistical significance was determined based on adjusted p-values after the Benjamini-Hochberg correction [40]. Then, multivariate data were reduced to two-dimensions and visualized by a canonical analysis of principal coordinates (CAP) using *CAPdiscrim* {BiodiversityR} function [41]. Random forest (RF) classification algorithm (number of trees = 500, mtry = 2) was implemented to test the ability of trace elements and stable isotope signatures to discriminate among fish belonging to different nursery areas [42]. Data was split into a training dataset (70%) and a testing dataset (30%), and this procedure was randomly repeated 1000 times. At each time, the rate of classification success (i.e., rate of correct predicted membership to nursery areas in which the fish were collected) was calculated, and mean values were extracted. Cohen´s Kappa (κ) statistic was also calculated, which is a method that accounts chance-corrected percentage of agreement between actual and predicted group memberships. Values of κ range from 0 to 1, where 0 indicates that the RF resulted in no improvement over chance, and 1 indicates perfect agreement [43]. Random forest was performed for each cohort, as well as for pooled data from both cohorts in the case of trace element data. For stable isotope data, and combined trace element and stable isotope data, pooled data from both cohorts was only used.

## Results

### Individual elements

**Fig 2:**
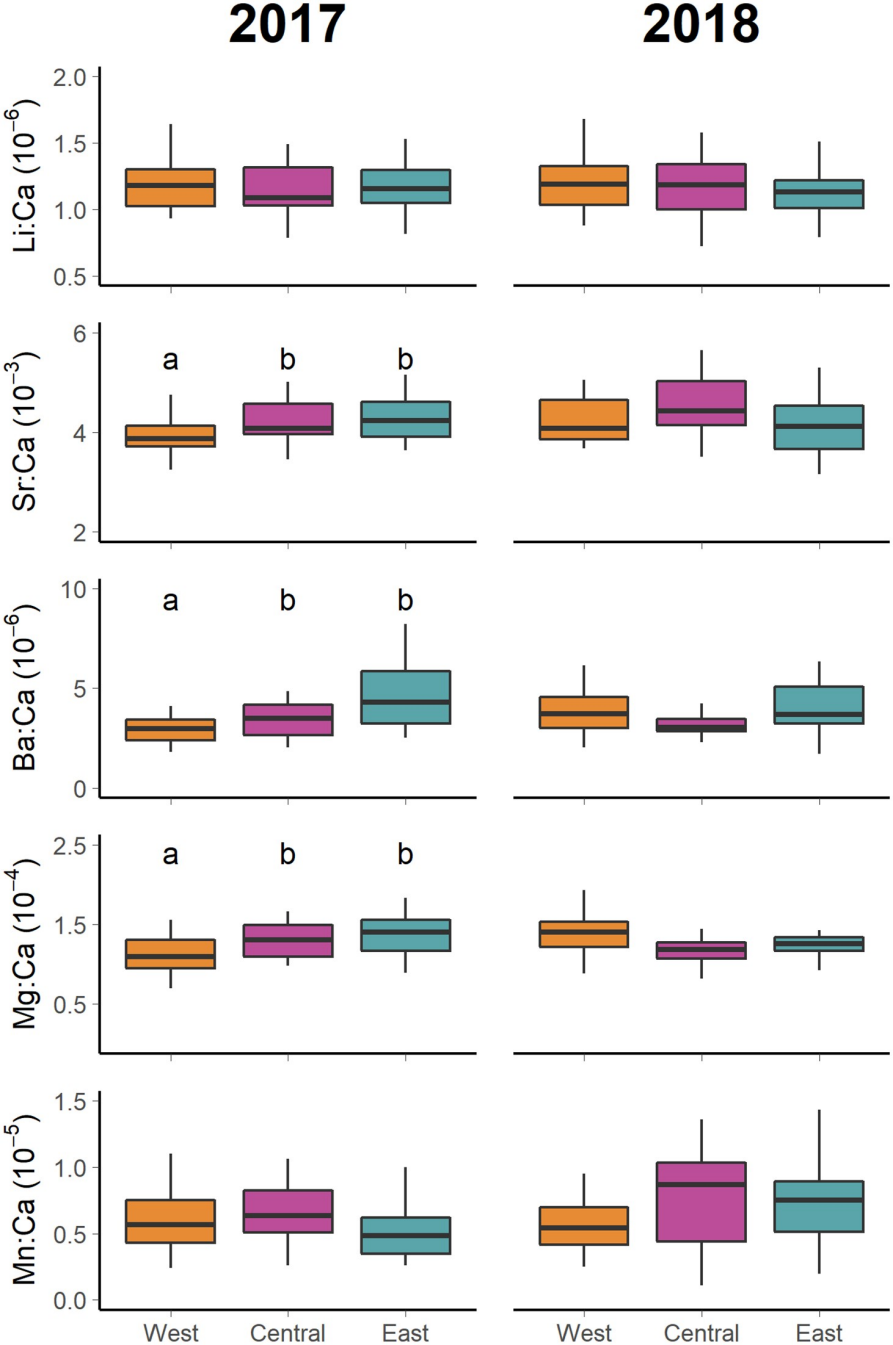
Trace element otolith composition of young-of-the-year (YOY) skipjack tuna (*Katsuwonus pelamis*) in the Indian Ocean. Boxplots compare otolith Li:Ca, Sr:Ca, Ba:Ca, Mg:Ca and Mn:Ca composition between nursery areas in the Indian Ocean in 2017 cohort (left panel), 2018 cohort (rigth panel). Letters identify significant differences (heteroscesadic anova, p<0.05) between group means. Inter quartile range (25th and 75th percentile) is shown by extent of boxes and error bars represent 10th and 90th percentiles.

Significant differences were detected in the chemical signatures of skipjack tuna collected from different nurseries, but also between cohorts in the case of trace element data (Table 3). The concentrations of Sr and Mn differed significantly between years, concentrations being higher in 2018 (Fig 2). Significant interactions between nursery and cohort were also detected for Ba, Mg and Mn, meaning that the pattern of variation was different among nurseries at each cohort (Table 3, Fig 2). Concentrations of Sr, Ba and Mg differed among nurseries in 2017, being lower for skipjack tuna collected from the West nursery (Fig 2). For fish belonging to the 2018 cohort no significant differences across regions were detected for any of the trace elements analyzed.

**Table 3:** Summary of two-factor PERMANOVA for the effect of nursery and cohort on individual and combined trace element data of skipjack tuna (Katsuwonus pelamis) otoliths.

Element	Source	df	F	P-value
Li	Nursery	2	0.13	0.879
	Cohort	1	0.11	0.746
	Nursery x Cohort	2	0.01	0.693
Sr	Nursery	2	5.06	0.011*
	Cohort	1	5.11	0.023*
	Nursery x Cohort	2	1.45	0.260
Ba	Nursery	2	5.35	0.007**
	Cohort	1	1.26	0.272
	Nursery x Cohort	2	4.99	0.011*
Mg	Nursery	2	1.97	0.122
	Cohort	1	0.37	0.541
	Nursery x Cohort	2	4.10	0.014*
Mn	Nursery	2	2.04	0.128
	Cohort	1	4.90	0.025*
	Nursery x Cohort	2	3.79	0.038*

Nursery area and cohort were fixed factors within the full factorial design. Significant effects are highlighted as follows;

*P<0.05,

**P<0.01.

**Fig 3:**
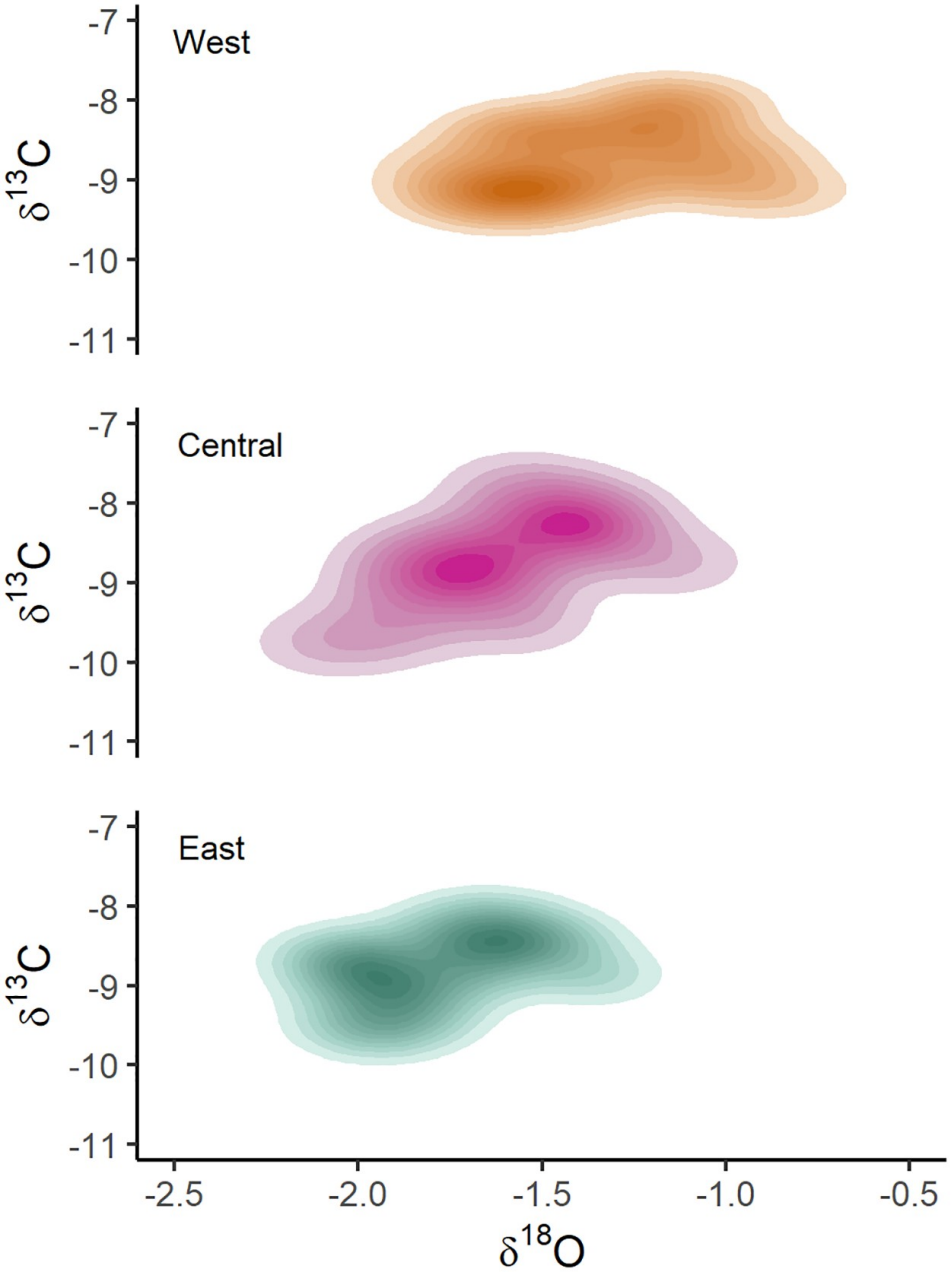
Contour plots of δ^13^C and δ^18^O values in otoliths of young-of-the-year (YOY) skipjack tuna (*Katsuwonus pelamis*) in the Indian Ocean. The composition of each nursery area is shown: West (orange), Central (purple) and East (green). Bivariate kernel density estimated at ten levels (10%, 20%, 30%, 40%, 50%, 60%, 70%, 80%, 90% and 100%).

Otolith δ^18^O values also varied among nurseries (anova, p<0.001), with significantly higher values found in the West nursery with respect to the central and eastern nurseries (Fig 3). No significant differences were detected in otolith δ^13^C composition between nursery areas.

### Multielemental signatures

**Fig 4:**
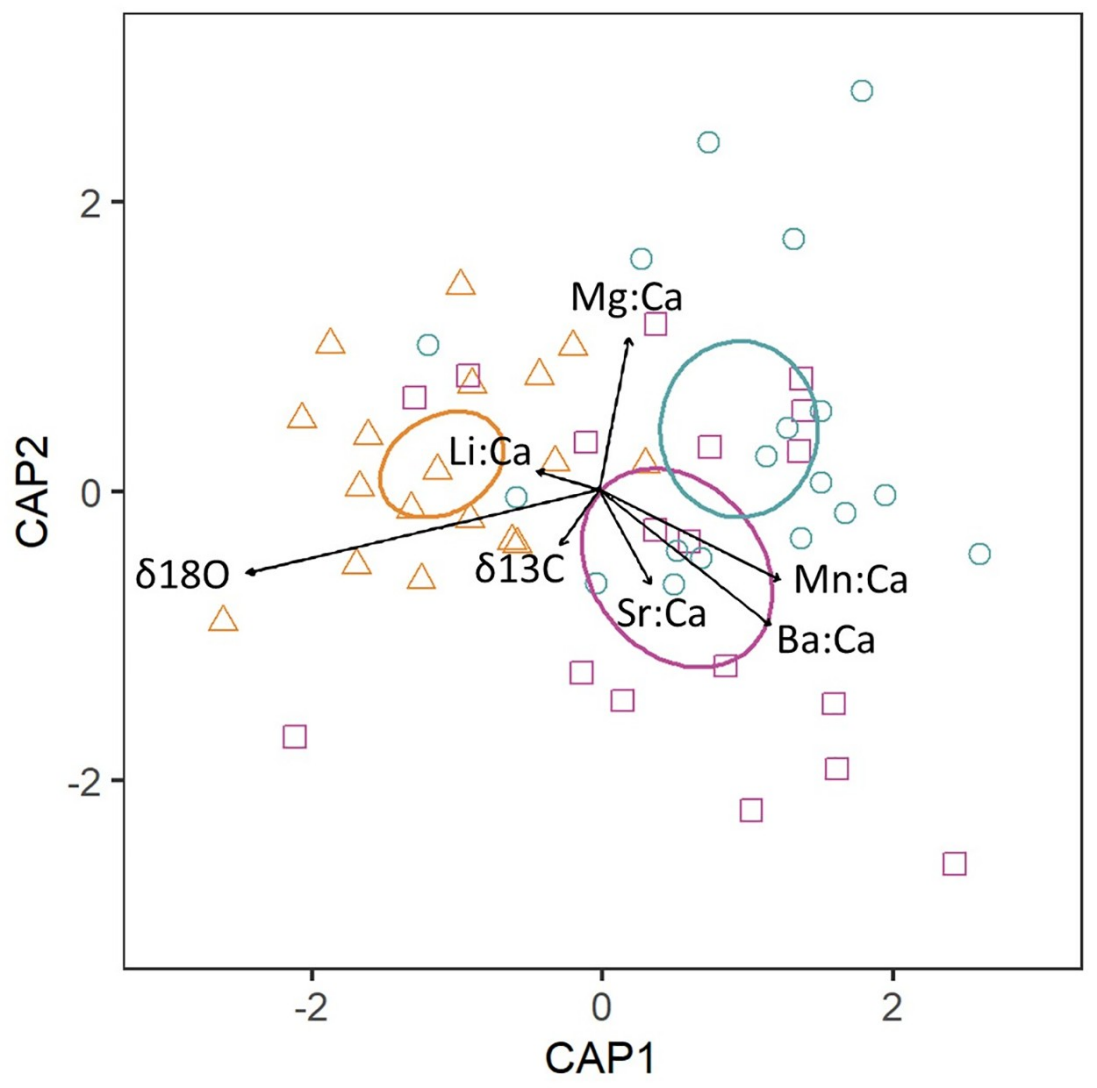
Canonical analysis of principal coordinates (CAP) variate plot of the multielemental chemistry of skipjack tuna (*Katsuwonus pelamis*) otoliths. Ellipses show 95% confidence around nursery means, shown as West (triangles, orange), Central (squares, purple) and East (circles, green).

When individual trace elements and carbon and oxygen stable isotopes were combined, significant regional differences were found among nursery areas (PERMANOVA, p = 0.005). Specifically, multielemental signatures from YOY skipjack tuna from the western nursery were differentiated from the central and eastern nurseries, as supported by posterior pairwise comparisons (Table 4, and Fig 4).

**Table 4:** Results of pairwise comparisons of multielemental (Li:Ca, Sr:Ca, Ba:Ca, Mg:Ca, Mn:Ca, δ^13^C and δ^18^O) signatures among three nursery areas in the Indian Ocean.

Nursery pair	Df	Sum of squares	F-statistic	R squared	P value	Adjusted p value	Significance
West vs Central	1	22.77	3.88	0.10	0.002	0.003	**
West vs East	1	27.77	4.55	0.11	0.001	0.003	**
Central vs East	1	8.60	1.13	0.03	0.335	0.335	

In the significance column, a star (*) means significant differentiation at <0.05 level and two stars (**) at <0.01 after adjusted p-value calculation with Benjamini and Hochenberg (BH) correction.

Classification accuracy was generally low regardless of the elemental combination used. Classification success using cohort pooled cohort trace element data was of 44% and κ = 0.16 (Table 5). Overall classification success of fish back to their nursery area was higher in 2017 (50%, κ = 0.33) than in 2018 (44%, κ = 0.15). Nursery specific signatures also varied considerably between cohorts. For YOY skipjack tuna from the West nursery classification accuracy decreased from 71% in 2017 to 49% in 2018, while for the Central nursery increased from 23% in 2017 to 40% in 2018. Cohort pooled classification accuracy of YOY skipjack tuna from East nursery remained similar (45% and 42% in 2017 and 2018, respectively).

**Table 5:** Random forest discriminant function analysis classification success (%) assigning young-of-the-year (YOY) skipjack tuna (*Katsuwonus pelamis*) to their nursery area in the Indian Ocean.

Element combination	Nursery	Both cohorts
Li:Ca, Sr:Ca, Ba:Ca, Mg:Ca, Mn:Ca	West	59
	Central	27
	East	39
	Overall	**44**
	κ	*0*.*16*
δ^13^C, δ^18^O	West	66
	Central	31
	East	47
	Overall	**51**
	κ	*0*.*44*
Li:Ca, Sr:Ca, Ba:Ca, Mg:Ca, Mn:Ca δ^13^C, δ^18^O	West	65
	Central	41
	East	46
	Overall	**51**
	κ	*0*.*40*

Results are shown for random forest using data from (1) trace elements (Li:Ca, Sr:Ca, Ba:Ca, Mg:Ca, Mn:Ca), (2) stable isotopes (δ^13^C and δ^18^O), and (3) trace elements and stable isotopes combined, and both cohorts pooled.

Carbon and oxygen stable isotopes provided higher classification success than trace elements alone (51%, κ = 0.44, Table 5). The combination of trace elements and stable isotopes did not improve the overall classification accuracy with respect to the use of carbon and stable isotopes alone (51%, κ = 0.40). However, nursery specific classification did vary, YOY from the West nursery were better classified with stable isotopes alone, while for YOY from the Central nursery greater classification accuracy was obtained when stable isotope data was combined with trace element data (Table 5, Fig 4). Nevertheless, samples from the Central and East nurseries were often misclassified (<50%), regardless on the elemental combination used.

## Discussion

Otolith chemical fingerprints are powerful discriminators of groups as long as differences exist, but of negligible value when differences cannot be detected, as the absence of difference does not necessarily imply that groups own a common origin [20]. The high overlap in the otolith chemical signatures of YOY from the central and eastern nurseries, may be at least partly explained by the homogeneity in the physicochemical properties of ambient water fish were exposed to during their early life. Depending on the period, the regions of Maldives (Central) and eastern Indian Ocean are quite homogeneous in terms of sea surface temperature (SST), salinity (SSS), and dissolved oxygen (DO) [44, 45]. Another potential reason to the observed overlap of the chemical signatures, is that important transoceanic mixing may have occurred prior to capture. However, it is unlikely that transoceanic migrations occur before 4 to 6 months of life. To our knowledge, transoceanic movements of skipjack tuna within the first 6 months have not been documented, since tagging studies are generally not available for these size ranges [11]. Moreover, skipjack tuna predominantly shows diffusion type movements rather than long longitudinal migrations, as they move towards colder and richer subtropical areas adjacent to their spawning areas, where they can temporarily found higher productivity and prey availability than in warm equatorial waters [8, 10]. Besides, the sub-mesoscale activity present in the nursery areas can minimize long-distance dispersal, by retaining the larvae near their spawning area [46, 47]. For all the above, we think that the observed results in the chemical signatures of YOY skipjack tuna are more likely attributed to the fact that biochemical properties of the water masses were relatively homogeneous during the early life of the fish, leading to a low discrimination capacity among fish captured in different nursery areas.

Strong interannual effect was detected in the otolith trace element signatures of fish belonging to two different cohorts. While significant differences were detected among nurseries in Sr, Ba and Mg concentrations in 2017, differences were not significant in 2018. Barium and strontium are incorporated into the otolith via Ca substitution, and may be appropriate markers of environmental history [48–50]. Mg incorporation into the otolith by contrast, has been related with metabolic activity [51, 52]. Due to sampling constrains, YOY skipjack tuna were hatched during different year periods (S1 Fig). North of 10°S the Indian Ocean is characterized by two seasons with distinct wind regimes, the monsoon system, which drives the ocean circulation and climate in the tropics and northern subtropic region of the Indian Ocean [53, 54]. The southwest monsoon, also known as summer monsoon, takes place from June to October with a maximum of intensity during the months of July, August, and September. During this season there is little difference in rainfall intensity within the equatorial region, while temperatures are colder west of 60 °E due to the coastal upwelling that takes place off Somalia and Oman Coasts [44, 45, 55]. The northeast Monsoon, or the winter monsoon, takes place between December and April with a maximum of intensity in the months of January, February, and March. Upwelling events take place off northwest Australian shelf and in open ocean at 5–10 °S region [53]. During the winter monsoon waters off Somalia are colder (26.5°C), whereas SSTs are above 28°C over the rest of the equatorial region [45]. Besides, during this season rainfall intensity is stronger in the west south (4.5°S) of the equator off Madagascar and off Sumatra [55]. During the transition periods between the monsoons (i.e., May and November), a strong equatorial downwelling occurs. For skipjack tuna belonging to the 2017 cohort, those collected from the West nursery were estimated to be hatched during the summer monsoon, while those from Central and East nurseries were considered to be hatched during the winter monsoon and the autumn intermonsoon periods. All skipjack tuna belonging to the 2018 cohort were estimated to be hatched within the summer monsoon period and the two intermonsoons. As seasonal climatology strongly influences the biochemical variability throughout the northern Indian Ocean, it may be possible that observed regional differences in Sr, Ba and Mg in 2017 are due to the seasonal changes in water physicochemical properties that could have masked regional differences [56, 57].

Otolith δ^18^O signatures were significantly higher from individuals of the West nursery than the Central and the East nurseries. The isotopic composition of ambient seawater is dependent on evaporation, and largely controlled by salinity [58]. As otolith δ^18^O is incorporated into otolith aragonite close to equilibrium with seawater, the isotopic composition of otolith reflects seawater δ^18^O values, being inversely correlated with ambient seawater temperature [59, 60]. Otolith composition of YOY skipjack tuna from the Indian Ocean followed the expected trend for δ^18^O signatures, presenting higher values in the West (expected due to lower water temperatures, under the influence of the seasonal Somali upwelling) and decreasing towards the east (expected due to higher water temperatures, as confirmed by the overall sea surface temperature pattern in the Indian Ocean). Similar trends have been found in a preliminary study for yellowfin tuna from the Indian Ocean [61]. No differences were detected in otolith δ^13^C signatures between fish captured in the three different nursery areas. Otolith δ^13^C can be a proxy for metabolic rate in fish, and is influenced by the environment (dissolved inorganic carbon in water, DIC) and diet [62, 63]. The observed absence of difference in δ^13^C signatures followed the expected trend for surface water carbon isotope composition, which is relatively homogenous within the equatorial Indian Ocean [64], and therefore providing little value for stock discrimination.

The overall low regional variability in chemical signatures from skipjack tuna resulted in a low classification of YOY individuals to the three nursery areas analyzed in the Indian Ocean. To date, there are no studies aiming to retrospectively determine skipjack tuna to their nursery origin based on otolith chemical signatures. However, studies done with other tuna species inhabiting tropical environments (e.g. yellowfin and bigeye) reported higher classification accuracies of fish back to their nursery area than those reported in this study [24–26, 65]. The use of stable isotopes alone was sufficient for nursery discrimination of yellowfin tuna in the Pacific Ocean, whereas trace elements proved to be more efficient for discriminating among yellowfin nursery areas in the Atlantic and Pacific and Indian Oceans [24, 26, 65]. Here, the use of carbon and oxygen stable isotope values alone, proved to be effective to differentiate skipjack tuna from the western nursery, but the high overlap in the chemical signature of skipjack tuna from the Central and East nurseries resulted in an overall low classification. The use of trace element data or the combination of stable isotope and trace element data did not improve the overall classification success. It is also possible that differences in classification success between trace elemental ratios and stable isotopes is due to the different the time period of the otolith represented by each tracer (i.e. ~day 13 to 15 for trace elements vs. 4–6 months for stable isotopes) [24]. One possible solution would be to analyze with LA-ICPMS the integrated signal of an ablation square that corresponds to the same otolith portion milled for stable isotopes analyses (i.e. ~4–6 months of life). However, this data should be interpreted with caution as it has been proved that ontogeny strongly influences trace element uptake into the otolith [52, 66].

## Conclusions

Overall, the differences in the chemical signature of YOY skipjack tuna from the three nursery areas in the Indian Ocean were not strong enough to describe a reference baseline for each nursery that allows the assignment of older individuals to their origin nursery. Temporal differences in the physicochemical characteristics of the Indian Ocean seem to strongly influence otolith trace element composition. Considering also that classification success for models based on otolith trace element data compared to those using only otolith stable isotopes was lower, the effort and cost to incorporate elemental ratios into Indian Ocean skipjack tuna otolith chemistry baseline delineation may not be worthwhile [25]. Until there is a proper understanding of the stock structure of skipjack tuna, the uncertainty in relation to the response of this stock to management decisions adopted considering a single stock will be maintained. Further research on skipjack population structure using otolith microchemistry should rely on younger (<4 months) individuals to reduce the possibility of movements between nursery areas and consider temporal stratification of sampling so that seasonal differences in oceanography can be disentangled from potential regional differences. Finally, an holistic approach may provide a more accurate overview to properly define management units, as single technical approaches may not be sufficient to delineate complex stock structures [67, 68]. Recent studies combining different stock delineation techniques (e.g. otolith microchemistry, genetics, biophysical models…), proved to be more effective to fully understand the spatial ecology of highly migratory fish species, and, hence increased the resolving power of stock discrimination [69–71]. For instance, including passive drift trajectory simulations may also be helpful to understand potential patterns of skipjack larval dispersal in the Indian Ocean [72]. The combination of otolith chemistry data coupled with genetic analyses can increased details on connectivity patterns, as both techniques provide information on complementary timescales (individual for otolith chemistry, and evolutionary for genetics), unravelling otherwise hidden patterns [73]. Ultimately, a better understanding of the stock structure and spatial connectivity of skipjack tuna in the Indian Ocean will be essential to implement and enforce management strategies that ensure long-term sustainable fisheries of this important species.

## Supporting information

S1 Fig Back-calculated hatch period. Frequency histograms show the distribution of estimated hatching dates (year_month) of young-of-the-year (YOY) skipjack tuna (*Katsuwonus pelamis*) collected in three nursery areas West (orange), central (purple) and East (green) of the Indian Ocean.
(TIF)

S2 Fig Chemical analyses location in the otolith. Transverse section of a 33 cm FL skipjack tuna (*Katsuwonus pelamis*) otolith. Approximate location of laser ablation spot for trace element analyses (left) and the MicroMill drilling path used for stable isotope analyses (right) are shown.
(TIF)

S1 Table Raw data. Values of trace element ratios (Ba:Ca and Sr:Ca, ppm) and stable isotopes (δ^13^C and δ^18^O, ‰) for the young-of-the-year (YOY) skipjack tuna (Katsuwonus pelamis) analysed in this study.
(CSV)
