# Supplementary figures and images for: Otolith chemical fingerprints of skipjack tuna (Katsuwonus pelamis) in the Indian Ocean: First insights into stock structure delineation
Source: PLoS One. 2021 Mar 29;16(3):e0249327. doi: 10.1371/journal.pone.0249327 (PMC8006990; doi:10.1371/journal.pone.0249327)

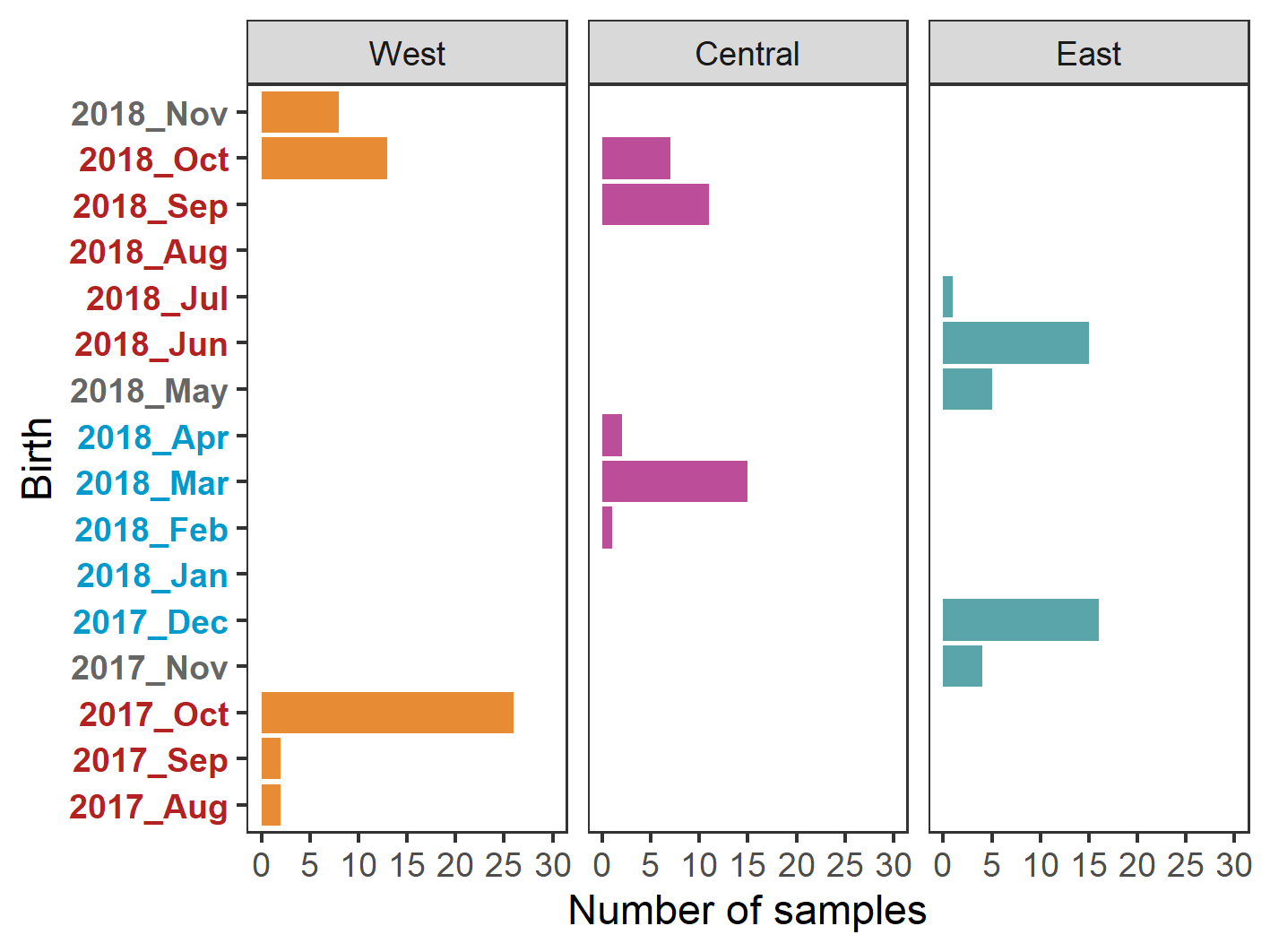

Supplement: S1 Fig — Frequency histograms show the distribution of estimated hatching dates (year_month) of young-of-the-year (YOY) skipjack tuna (Katsuwonus pelamis) collected in three nursery areas West (orange), central (purple) and East (green) of the Indian Ocean. (TIF) [file pone.0249327.s001.tif]

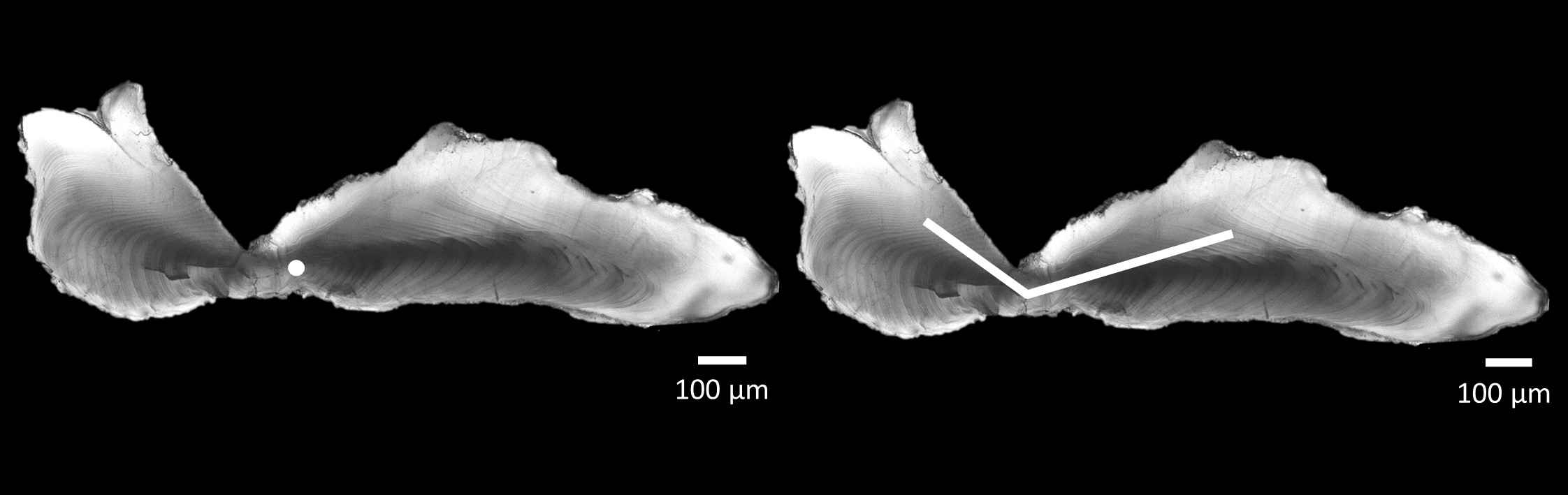

Supplement: S2 Fig — Transverse section of a 33 cm FL skipjack tuna (Katsuwonus pelamis) otolith. Approximate location of laser ablation spot for trace element analyses (left) and the MicroMill drilling path used for stable isotope analyses (right) are shown. (TIF) [file pone.0249327.s002.tif]
